# Hemodynamics of Cerebral Aneurysms: Computational Analyses of Aneurysm Progress and Treatment

**DOI:** 10.1155/2012/782801

**Published:** 2012-02-19

**Authors:** Woowon Jeong, Kyehan Rhee

**Affiliations:** Department of Mechanical Engineering, Myongji University, 38-2 Nam-Dong, Yongin-Si, Kyunggi-Do 449-728, Republic of Korea

## Abstract

The progression of a cerebral aneurysm involves degenerative arterial wall remodeling. Various hemodynamic parameters are suspected to be major mechanical factors related to the genesis and progression of vascular diseases. Flow alterations caused by the insertion of coils and stents for interventional aneurysm treatment may affect the aneurysm embolization process. Therefore, knowledge of hemodynamic parameters may provide physicians with an advanced understanding of aneurysm progression and rupture, as well as the effectiveness of endovascular treatments. Progress in medical imaging and information technology has enabled the prediction of flow fields in the patient-specific blood vessels using computational analysis. In this paper, recent computational hemodynamic studies on cerebral aneurysm initiation, progress, and rupture are reviewed. State-of-the-art computational aneurysmal flow analyses after coiling and stenting are also summarized. We expect the computational analysis of hemodynamics in cerebral aneurysms to provide valuable information for planning and follow-up decisions for treatment.

## 1. Introduction

Aneurysm is a vascular disease characterized by local dilatation of arterial walls. Aneurysms are frequently observed in the intracranial space and exhibit fusiform or saccular shapes. Some of aneurysms may grow, and rupture of cerebral aneurysms causes intracranial hemorrhage, which is associated with high mortality and morbidity [1–3]. In order to prevent the rupture of aneurysms, interventional thromboembolization treatment via the endovascular insertion of coils and stents may be applied as a prophylactic treatment. The recent development of neuroradiological and diagnostic imaging techniques has enabled aneurysms to be detected more frequently. Since much less than 1% of cerebral aneurysms rupture on an annual basis [4, 5], the demand for accurate prediction of aneurysm growth and rupture is increasing in order to select appropriate and immediate endovascular treatment.

 The initiation, progression, and rupturing of aneurysms are related to the arterial wall remodeling: it is believed that they are all related to the complex interactions between biochemical and biomechanical factors. Pathological vessel wall remodeling involves various enzymes and proteins related to degeneration, inflammation, and repair: their expressions in arterial walls can be affected by hemodynamics. Blood flow imposes mechanical stress on the vessel wall, which may stimulate the functions of endothelial cells, affect the structural integrity of the endothelium, and affect the transport of various cells and enzymes in the blood stream to the endothelium. Therefore, hemodynamic forces and flow characteristics, such as recirculation [6], secondary flow [7], and jet impingement [8], are considered to be major mechanical factors related to the genesis and progression of vascular diseases. Among the hemodynamic parameters, wall shear stress (WSS) has been studied extensively, since endothelial cells actively sense and respond to WSS. In their recent review, Nixon et al. [9] clearly summarized the role of WSS in cerebral aneurysms and atherosclerosis.

**Figure 1:**
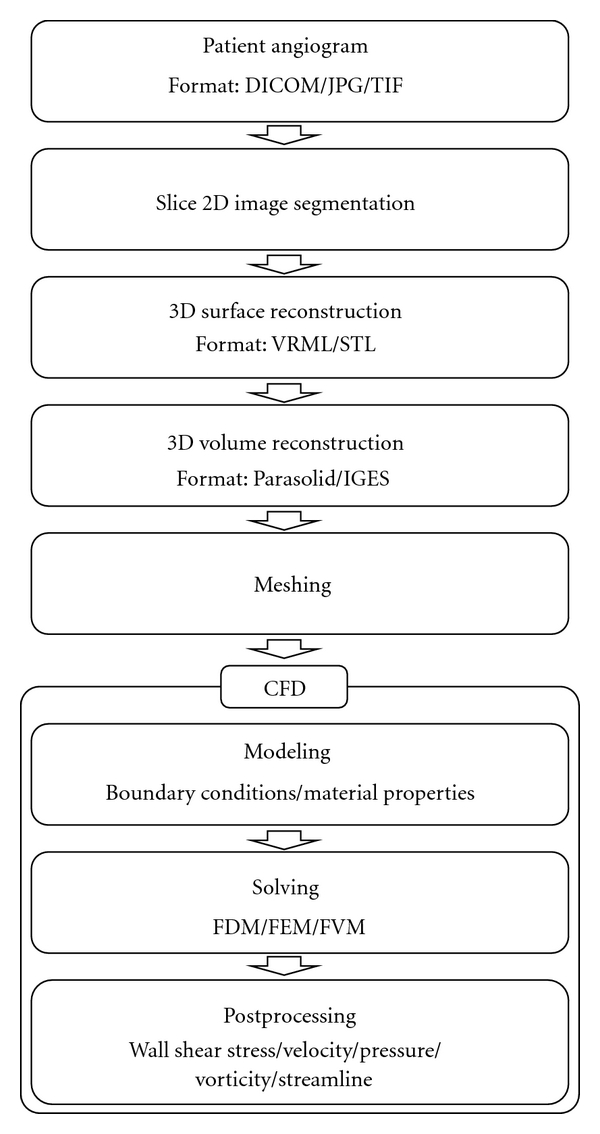
Flowchart of the computational hemodynamic analysis procedure based on patient-specific angiogram.

 Recent progress in medical imaging technology and improvements in computer equipment have enabled computational fluid dynamic (CFD) analysis to predict the hemodynamics of aneurysms with increased accuracy and reliability. Angiography image data can be converted to the three-dimensional (3D) vessel geometric data for computer simulation; therefore, CFD analysis based on real aneurysm geometry has been progressed in recent years [10–14]. The basic process of computational hemodynamic analysis using a patient angiogram is illustrated in Figure 1. The sliced cross-sectional lumen images of a patient's vasculature are obtained using various imaging modalities, such as magnetic resonance angiography (MRA), computed tomography angiography (CTA), and 3D rotational angiography. The lumen of each cross-section image can be segmented and the luminal surface is reconstructed using splines, contour tilting, or other interpolation methods. Two-dimensional segmentation is inaccurate when the vessel axis is not perpendicular to the cross-sectional surface. Furthermore, manual automatic segmentation is operator dependent while automatic segmentation using threshold may yield topological defects and inaccuracies for the image with inhomogeneous image intensities. The state-of-the-art models for unsupervised full three-dimensional segmentation have been developed [15]. A region growing segmentation with automatic thresholding [16] and a component-based approach using a deformable model [17] are described in Cebral et al. [15] in detail. Based on the reconstructed surface contour, 3D solid-volume models are constructed using commercial 3D computer-aided design (CAD) programs. The reconstructed 3D volume models are divided into the computational grids by a meshing program. Some commercial software for preprocessing is outlined in Table 1. The governing equations of a flow fields are solved using various numerical schemes, such as the finite differential method (FDM), finite element method (FEM), finite volume method (FVM), and lattice Boltzmann method (LBM). Various commercial CFD software programs including Fluent and Ansys CFX (ANSYS, Inc., Canonsburg, PA, USA), ADINA (ADINA R&D, Inc., Watertown, MA, USA), COMSOL (COMSOL, Inc., Burlington, MA, USA), CFD-ACE (ESI Group, Paris, France), Flow-3D (Flow Science, Inc., Pasadena, CA, USA), and STAR-CD (CD-adapco, Melville, NY, USA), are available. Calculated flow field variables can be rearranged in order to show various flow characteristics of interest using postprocessing procedures.

Patient-specific CFD analysis involves imaging data process, mesh construction, computational calculations, and postprocessing. In order to avoid difficulties associated with manual and time-consuming works of using commercial CFD packages, efforts of developing in-house CFD codes have been made. The whole pipeline from medical images to flow calculations has been developed in order to eliminate manual intervention and editing of data processing. Cebral et al. [10, 15, 18, 19] developed the pipeline for simulation based hemodynamics. It consists of vessel reconstruction, unstructured grid generation, numerical solver, and postprocessing. Proper boundary conditions and material properties are imposed on the blood vessel models. Patient-specific inflow data provide important information which determines the accuracy of CFD calculations. In vivo patient-specific inflow boundary conditions can either be obtained through invasive measurement using a catheter or medical imaging. Karmonik et al. [20–22] measured blood flow waveform in cerebral arteries by phase contrast MRI, and provided the measured blood flow profile at the inlet boundary conditions for CFD simulation. They successfully simulated blood flow in cerebral arteries using 3D digital subtraction angiography and phase contrast MRI. Specifying outflow boundary condition also requires careful attentions, and the appropriate impedances of the distal vasculature for multiple outlets should be provided.

If the hemodynamic factors affecting aneurysm etiology are elucidated, CFD analysis based on the patient-specific images data will provide a better understanding and diagnosis of aneurysm progress and rupture. The performance of endovascular stents and coils can be evaluated by analyzing aneurysmal flow alteration as a result of interventional treatment. Furthermore, prediction of blood flow using CFD analysis can be used to plan interventional therapies. In this paper, we review recent computational hemodynamic studies on cerebral aneurysm initiation, growth, and rupture, as well as computational studies on aneurysmal flow alterations induced by interventional treatment with coils and stents.

## 2. Aneurysm Initiation

 Most cerebral aneurysms are observed at arterial bifurcations and branches or at the outer walls of arterial curvatures [23, 24]. The localized occurrence of cerebral aneurysms prompted the hemodynamic research on aneurysm formation. Since CFD analysis provides detailed hemodynamic information at bifurcations [25, 26] and the outer walls of curved arteries [27, 28], many studies have attempted to identify an appropriate hemodynamic parameter correlated with pathological aneurysm formation.

**Figure 2:**
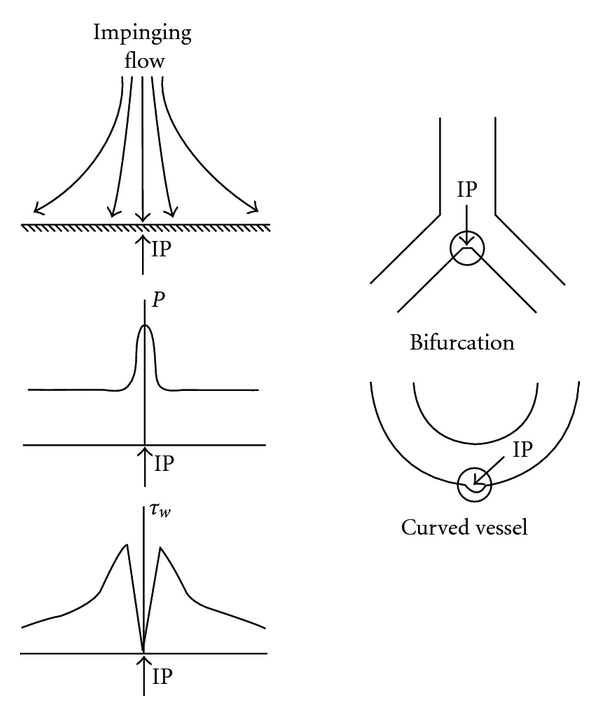
Schematic of pressure (*P*) and WSS (*τ*_*w*_) distribution near the jet impingement point (IP) of curved and bifurcated blood vessels.

Flow impingement on the apex of bifurcations and sharply curved vessels generates unstable helical flow patterns near the impinged wall. Repetitive flow impingement against the vessel wall under pulsatile flows may induce fatigue, potentially causing morphological and functional changes in the endothelium may occur in this region. The distributions of pressure and shear stress near the impingement point are shown in Figure 2. The local pressure increase at the impingement point is caused by the conversion of fluid kinetic energy to static wall pressure. Previous studies reported that the local pressure increases at arterial bifurcations and bends are less than 1-2% of intravascular pressure [29, 30]. However, high spatial pressure gradients may affect endothelial remodeling.

WSS near the impingement point is high (hundreds of dyne/cm^2^ [31]), and its spatial gradient is also very large. Previous studies using animal experiments have demonstrated that high WSS contributes to the genesis of cerebral aneurysms via degenerative changes in endothelium [32, 33]. Other studies have also shown that elevated WSS affects to the various degenerative changes of vessel walls [34–40].

Meng et al. [26] surgically created carotid bifurcations in a dog animal model and found that the spatial histological features of walls are correlated with hemodynamic variables calculated by CFD analysis. Their results show that the localization of destructive wall remodeling, which resembles the initiation of aneurysms, is correlated with a combination of high WSS and a high WSS gradient. Mantha et al. [37] constructed computational models of carotid artery lateral aneurysms based on the patient 3D angiography data, and performed CFD analysis was performed for the parent vessel prior to aneurysm formation. They found significant correlations between the temporal directional changes of WSS and the location of aneurysm formation.

Since the endothelium senses WSS and actively responds to mechanical stress [34–36, 38, 40], vascular remodeling that initiates aneurysm formation may be related to high WSS magnitude or high spatial and temporal variations in WSS. Although it is difficult to pinpoint the hemodynamic variables responsible for aneurysm initiation, complex flow fields near the flow impingement point, where high WSS magnitude, high temporal and spatial WSS gradients, and high pressure gradients are probably part of the prerequisite hemodynamic environment related to the initiation of cerebral aneurysms.

## 3. Aneurysm Growth

Histological studies have revealed degeneration of the intima and thinning of the media in the aneurysm vessel wall [39, 41]. Furthermore, various proteolytic enzyme secretions such as elastase [42] and matrix metalloproteinase [43] contribute to degenerative wall remodeling. Pathological wall remodeling processes, which are related to the secretion of various enzymes, along with the inflammatory response of endothelium [44] and apoptosis of smooth muscle cells [45, 46], may be affected by hemodynamics [47]. CFD studies have been performed to investigate the hemodynamic factors affecting aneurysm growth. Many CFD studies have been performed on the growth and rupture of aneurysms using not only the ideal curved and bifurcated blood vessel models [31, 48–51], but also aneurysm models based on real patient data obtained by MRI or CT imaging [13, 14, 52, 53].

Feng et al. [54, 55] simulated the deformation and growth of an aneurysm bleb of a curved vessel in a simplified cerebral artery model. They assumed that aneurysm growth was related to degeneration of the arterial wall caused by high WSS. Aneurysm formation was modeled as vessel wall deformation due to reduced wall stiffness using fluid solid interaction (FSI). The WSS distributions were calculated, and the Young's modulus of the arterial wall was assumed to be reduced if the WSS was larger than the threshold value. The drawback of their methodology is that the mechanical properties of an arterial wall could not be modeled as a simple function of WSS. Furthermore, arterial remodeling could not be simply modeled by using a vessel wall expansion model based on FSI, and the temporal progression of aneurysm growth was not considered. Boussel et al. [56] calculated the WSS of the intracranial aneurysm on the basis of the patient's MR angiography data, and correlated WSS with aneurysm growth by using MR images at 2 different time points (mean 16.4 ± 7.4 months between the 2 time points): they found a significant correlation between low time-averaged WSS and aneurysm growth.

Atherosclerotic vascular wall changes due to low and oscillatory WSS have been studied extensively, and the role of hemodynamics on atherogenesis is well established [57–59]. Although vessel wall remodeling in aneurysm growth is different from atherosclerotic wall remodeling [46, 60], inflammatory responses due to disturbed flow or decreased WSS may affect the aneurysm growth and rupture via degenerative vascular wall remodeling. Low WSS (<4 dyne/cm^2^) causes endothelial proliferation [61] and apoptosis [62]. Moreover, low WSS induced by slow flow recirculation in the complex flow fields in the aneurysmal bifurcations [53, 63] is positively correlated with aneurysm growth. One study found that the WSS of the aneurysms is significantly lower than that of the surrounding vasculature [53]. Furthermore, a relationship between local aneurysm growth and areas of low WSS at the intraluminal surface was found using patient aneurysm models [53, 56].

The complex flow patterns accompanying low and oscillating WSS may be correlated with aneurysm growth. Slight out-pouching of the aneurysm wall may induce a disturbed flow region, and aneurysm growth can be accelerated due to low and oscillatory WSS in the expanded blood vessel via a positive feedback mechanism. Vascular wall remodeling maintaining the WSS at a constant level favors the high WSS hypothesis of aneurysm growth. However, it cannot explain aneurysm growth, because intracranial arteries have different vessel wall structures from the arteries in which arterial remodeling is observed [64–67].

## 4. Aneurysm Rupture

Aneurysm rupture occurs when wall tension exceeds the mechanical strength of wall tissue. Wall tension is proportional to the intramural pressure and radius, and inversely proportional to the walls thickness of a spherical balloon. Therefore, high pressure, large aneurysm size, and thin wall increase wall tension. Local weakening of the aneurysm wall, which is characterized by thinning of the media and a lack of collagen fibers, is closely related to the pathological wall remodeling. Furthermore, the hemodynamic environment may affect the wall remodeling process. Therefore, hemodynamic forces, including high pressure and WSS, may directly influence wall breakage, while low WSS and disturbed flow patterns may affect the aneurysm wall weakening via wall remodeling over an extended period.

Strong flow impingement is suspected as a hemodynamic factor responsible for aneurysm rupture. Cebral et al. [52] performed a CFD study using 62 patient-specific models. They found out that flow patterns are more complex and the impingement jet is narrower in ruptured aneurysms compared to unruptured aneurysms. Recently, the same group extended the previous study by including 210 intracranial aneurysms in 128 consecutive patients and more hemodynamic characteristics were investigated [68]. A quantitative hemodynamic study showed that concentrated inflow jets, small impingement regions, complex flow patterns, and unstable flow patterns were correlated with a clinical history of prior aneurysm rupture. Therefore, strong inflow jets from the parent vessel to the aneurysm sac might provide a hemodynamic environment prone to aneurysm rupture. Blood impingement on the wall generates impact force and high WSS, which could affect the fatigue of the vessel wall. The site of flow impact includes locally elevated pressure and a high WSS gradient. The contribution of pressure elevation due to the impinging flow jet is small [31, 69], but the effect of impact force remains unknown. Shojima et al. [53] calculated the WSS in the human middle cerebral artery using CFD analysis. They reported that the spatially averaged WSS of the aneurysm region at the peak systole is significantly higher (approximately 2 times higher) in ruptured models than that in unruptured models. High spatially averaged WSS in the ruptured cases is due to the high WSS at the body or the neck of aneurysm caused by direct flow stream from a parent artery. Therefore, high WSS may not be correlated with aneurysm rupture. The WSS at the tip of the aneurysm, where it is more vulnerable to rupture, is lower in the ruptured cases. But their computational results may not detect secondary flows and other detailed features of the intraaneurysmal flow pattern because the computational vessel models reconstructed in their study are extremely truncated. Castro et al. [18] studied the influence of the upstream parent artery geometry on intraaneurysmal hemodynamics. They showed that the reconstructed models using the truncated parent vessel underestimated the WSS and shifted the impact zone to the neck. Therefore, further studies should be required to clarify the effects of low and high WSS on aneurysm rupture.

Complex unstable flow accompanying low and oscillatory flow may be responsible for vessel wall remodeling associated with rupture [70], since it is correlated with apoptotic wall remodeling [62]. Valencia et al. [71] showed that low WSS regions are larger in ruptured aneurysms than in unruptured ones. They also report a linear correlation between the average WSS on the aneurysm sac at peak systole with the area index, which was defined as the ratio between the aneurysm area and the artery areas. Lu et al. [72] performed CFD analysis using 3D reconstructed angiograms of both ruptured and unruptured cerebral aneurysms. In the ruptured group, the proportion of low WSS areas (<1.5 Pa) to the whole area of the aneurysm was significantly lower, while the oscillatory shear index was significantly higher. Therefore, large spatial and temporal variations in WSS within the complex unstable flow in the aneurysm sac might be related to aneurysm rupture.

Pressure may be a hemodynamic parameter influencing aneurysm rupture. Previous studies demonstrated that the complex flow pattern of the flow impingement around the aneurysm results in elevated pressure at the aneurysm [30, 48, 69], although it is very low compared to systemic pressure. A large pressure gradient along a wall near the impingement point may affect endothelial functioning and remodeling. Torii et al. [73, 74] studied the effects of high blood pressure on WSS in aneurysm models using image-based FSI modeling. Their results show that hypertensive blood pressure causes significant changes in WSS distribution on the aneurysm wall. Thus, hypertension may affect aneurysm wall damage.

Since aneurysm rupture is not correlated with the abnormally high local stress, a loss of wall integrity due to vascular wall remodeling and increased wall tension due to systemic pressure may be responsible for aneurysm rupture. Isaksen et al. [75] calculated wall tension and displacement using FSI for the elastic cerebral aneurysm wall. They show that the areas of maximum wall tension and displacement are located where aneurysms are most vulnerable to rupture. Local changes of wall thickness and mechanical property caused by aneurysm wall remodeling were not considered since they assumed a uniform aneurysm wall thickness. Local wall thickness and the properties of the aneurysm wall are hard to measure, which limits the estimation of wall tension.

## 5. Aneurysm Coiling

Until the early 1990s, surgical clipping was used to obliterate the aneurysm sac. However, endovascular treatments such as coil embolization and stenting have been used since the development of nonsurgical endovascular approaches. Coils packed into the aneurysm sac induce flow stasis and thrombus formation [76–78], and scar tissue forms as a result of the foreign body reaction completing the aneurysmal occlusion [76, 79, 80]. It is difficult to completely fill an aneurysmal cavity due to the complex shape of the aneurysm sac and coil compaction due to hemodynamic force. Incomplete embolization induces residual flow from the parent vessel and contributes to the recurrence of the aneurysm. Accordingly, hemodynamics after coil embolization is a highly relevant factor for predicting the recurrence and regrowth of aneurysms.

Byun and Rhee [81] studied hemodynamic changes due to partially blocked lateral aneurysms, and investigated the effects of aneurysm shape and parent vessel geometry using CFD analysis. A computer simulation of a terminal aneurysm was also performed, and the effects of coil density were explored [76]. Since aneurysm wall remodeling and flow stasis may be important, aneurysmal WSS was investigated taking fluid (blood) and solid (vessel wall) interactions into consideration [82]. In these analyses, coils were modeled as a solid material (block element); these analyses might be valid for completely thromboembolized coils.

In order to investigate the flow inside the coil, the coil was modeled as a porous medium. CFD analysis was performed using aneurysm images of the real patient, and the influence of multiple coils on intra-aneurysmal hemodynamics was investigated [83, 84]. Porous medium modeling might be useful in the early stage of coil insertion, but the thrombus formation inside of a coil bundle should be modeled during the embolization process after endovascular treatment. Computational simulation of the blood clotting inside the idealized lateral aneurysm model was performed on the basis of the viscosity model defined as a function of both residence time and clotting fluid concentration [85]. However, further refinement of the clotting model is required, since its validation is incomplete.

The interaction between hemodynamics and the coil is the major physical cause of coil compaction and dislocation, which induce the recanalization and growth of the aneurysm. The interaction between coils and flow has been simulated. One application of these simulations is to simulate coil deployment during the initial stage of coil intervention. The results from such simulations may help provide patient-specific guidelines for coil selection and predict postoperative prognosis. Recently, Wei et al. [86] simulated coil deployment and deformation while considering reactive fluid force, and computed flow fields for coil filled and coil free domains. Since simulations of aneurysmal hemodynamics in the interaction between coils and flow are limited, further studies should be performed to elucidate the role of hemodynamics on coil compaction and dislocation.

## 6. Aneurysm Stenting

 Stents have been used as scaffolds to keep coils in the cavities of fusiform and wide-neck aneurysms. At present, it is believed that stents alone can be used to reduce the flow from the parent artery and to thromboembolize the aneurysm sac. Although thromboembolization with stents may be less effective than that with coils, the advantages of stenting include the ability to stabilize the aneurysm without touching the aneurysm sac and to block the aneurysm neck by inducing neointima formation over the stent surface. Predicting the hemodynamic changes after stenting could be helpful for designing and selecting stents effective for aneurysm embolization.

 Flow patterns, vorticity, slip velocity, and WSS are relevant hemodynamic parameters affecting the thromboembolization efficiency of stents. Flow visualization methods have been used in aneurysm phantom models to elucidate the effects of stent porosity [87–90]. Quantitative experimental methods using particle image velocimetry [91, 92] and laser Doppler velocimetry [93, 94] have also been used to evaluate alterations in hemodynamics according to different vessel geometries and stent designs.

 Computational analyses have been performed to elucidate the flow field alterations caused by stents and to evaluate their ability to divert flow and induce stasis inside of aneurysmal cavities [95, 96]. Patient-specific aneurysm models have also been analyzed to elucidate the flow alterations caused by stenting [11, 70, 97–102]. Recently, Cebral et al. [103] performed CFD analysis on the cerebral aneurysms which are apparently successfully treated using a stent but have been complicated by later aneurysm hemorrhage. They showed that flow diversion stent can cause intra-aneurysmal pressure increase, which can potentially lead to rupture. Among the challenges in the CFD simulation of stented aneurysms, there are major difficulties related to the meshing of stents due to the scale differences between the artery and stent strut thickness. Some adaptive embedding techniques have been developed to resolve such meshing difficulties [104–106]: vessel walls are treated with body-fitted unstructured grids, and stents are embedded in the grids; furthermore, adaptive meshing refinement is performed near the stents.

The flow of stented lateral and terminal aneurysms have been simulated by modeling a stent as a porous medium [107]. In this study, a rough model of stent geometry can reproduce flow features quantitatively as well as qualitatively. LBMs considering velocity reduction caused by stent implantation in advance using the stent positioning effect have also been developed to predict the thromboembolization of stented aneurysms [108–110]. The simulation of stent deployment is also challenging. In some models, an elastic cylindrical support surface is generated along the parent vessel that deforms until it comes into contact with the vessel wall [104, 111]. The inlet and outlet boundary conditions, as well as the parent vessel wall elasticity before and after the stenting, should be specified accurately. Further studies are required to model stent deployment as well as vessel wall and flow field alterations due to stenting, neointima formation along the stent, and thromboembolization modeling.

## 7. Discussion and Conclusions

Experimental hemodynamic studies have been performed using various cerebral aneurysm models in order to investigate the role of fluid dynamics in the etiology of aneurysms [48, 94, 111–115] and endovascular treatment [31, 105, 116]. These studies have attempted to find correlations between complex hemodynamic variables and cerebral aneurysm formation, growth and rupture with idealized model geometries. Hemodynamic alterations after stenting and coiling are also observed in in vitro models [81, 90] as well as in surgically created aneurysms in animal models [117]. Since the details of flow patterns are significantly affected by vessel and aneurysm geometries, realistic vascular geometry is believed to be one of the most important factors for precisely analyzing flow in aneurysms and arteries.

Recently, detailed patients' vessel geometries have become available as a result of the progress of high-resolution angiography techniques. Moreover, and the recent advances in information technology have enabled the prediction of hemodynamics in patient-specific blood vessels using CFD analysis. Difficulties in accurately determining blood vessel geometry from patient angiograms are related to the segmentation of the lumen, defining the calculation domain, and computational mesh formation. Since these difficulties require a great deal of manual, time-consuming, and operator-dependent work to be overcome, efforts for developing in-house codes for patient-specific CFD analyses have been made.

 Other difficulties in CFD analysis are related to defining the material properties and boundary conditions for realistic in vivo arterial blood flow. The consideration of the rheological viscosity characteristics of blood and vessel wall elasticity in arterial blood flow analysis has remained controversial over the last few decades. Even though non-Newtonian viscosity characteristics are not important for analyzing blood flow in large arteries [11, 118–121], they may influence the WSS distributions in aneurysmal flow [10]. The implementation of non-Newtonian viscosity laws in CFD calculations requires relatively insignificant efforts and computational loads, although validating the viscosity law in complex flow fields may be difficult. Taking the wall elasticity of arteries into consideration in computational analysis requires additional information on vessel radial wall motion, local wall properties, and pressure waveforms—all of which are difficult to obtain. CFD analysis using dynamic angiography has been attempted [122–124] to define radial vessel wall motion. Specifying outflow boundary conditions also requires careful attentions, and the appropriate impedances of the distal vasculature for multiple outlets should be provided.

Even though accurate vessel geometry can be obtained using high-resolution medical imaging modalities and hemodynamic analysis can be performed with the help of state-of-the-art computing techniques, the application of hemodynamic parameters for predicting aneurysm growth and rupture is still limited. Although information regarding vessel wall structure is very useful, it is difficult to obtain in vivo information of aneurysm wall structures. A vascular wall-remodeling model based on cell mechanobiology has been developed on the basis of FSI [125–127]. In this particular growth and remodeling model, hemodynamic analysis provides the wall tractions for wall mechanics computation to refine wall properties and geometries. The solution iterated between fluid and solid wall mechanics, and stress mediated-growth and aneurysmal remodeling have been analyzed. Modeling the changes in properties, configurations, and mass fractions of the aneurysmal wall due to mechanical stress requires further study [49, 125, 128]. Moreover, difficulties related to adaptive mesh generation [129–131] and boundary conditions for describing wall traction should be considered when computationally analyzing vascular wall remodeling.

Patient-specific hemodynamic simulations would provide valuable information for planning and follow-up decisions in cerebral aneurysm treatment. Additional effort is required to develop fast and accurate computational methodologies using high-resolution medical images to apply CFD simulation in cerebral aneurysm diagnostic and intervention planning tools. In addition, fundamental mechanobiological studies on the effects of hemodynamics on aneurysm growth, rupture, and thromboembolization should be performed in order to refine and complete the computational modeling.

## Figures and Tables

**Table 1:** Some commercial preprocessing software for patient-specific CFD analysis and its role.

Software	Slice 2D image segmentation	3D surface reconstruction	3D volume reconstruction	Meshing
3DMax (Autodesk Inc., San Rafael, CA)	Yes	Yes	Yes	No
3D-Doctor (Able Software Corp., Lexington, MA)	Yes	Yes	Yes	No
Mimics (Materialise Group, Leuven, Belgium)	Yes	Yes	Yes	No
Insight Toolkit (Kitware Inc., Clifton Park, NY)	Yes	Yes	Yes	No
3D Slicer (MIT, Boston, MA)	Yes	Yes	Yes	No
SolidWorks (Dassault Systems, Concord, MA)	No	No	Yes	No
Pro-Engineer (PTC, Needham, MA)	No	No	Yes	No
CATIA (Dassault Systems, Velizy-Villacoublay, France)	No	No	Yes	No
3-matic (Materialise Group, Leuven, Belgium)	No	No	Yes	Yes
Hypermesh (Altair Engineering Inc., Troy, MI)	No	No	Yes	Yes
Gambit (ANSYS Inc., Canonsburg, PA)	No	No	Yes	Yes
ICEM-CFD (ANSYS Inc., Canonsburg, PA)	No	No	Yes	Yes
Gridgen (Pointwise Inc., Fort Worth, TX)	No	No	Yes	Yes
